# Identification of Damage on Sluice Hoist Beams Using Local Mode Evoked by Swept Frequency Excitation

**DOI:** 10.3390/s21196357

**Published:** 2021-09-23

**Authors:** Qingyang Wei, Hao Xu, Yifei Li, Li Chen, Drahomír Novák, Li Cui, Maosen Cao

**Affiliations:** 1 College of Mechanics and Materials, Hohai University, Nanjing 210098, China; weiqingyang@hhu.edu.cn (Q.W.); felix@hhu.edu.cn (Y.L.); 2 State Key Laboratory of Structural Analysis for Industrial Equipment, Faculty of Vehicle Engineering and Mechanics, School of Aeronautics and Astronautics, Dalian University of Technology, Dalian 116024, China; xuhao@dlut.edu.cn; 3 Donghua Testing Technology Co., Ltd., Taizhou 214500, China; chenli@dhtest.com; 4 Faculty of Civil Engineering, Brno University of Technology, 60200 Brno, Czech Republic; novak.d@fce.vutbr.cz; 5 College of Civil and Architecture Engineering, Chuzhou University, Chuzhou 239000, China; cuili1996@126.com

**Keywords:** hoist beam, damage detection, local primary frequency, local mode, local resonance response band

## Abstract

As a global vibration characteristic, natural frequency often suffers from insufficient sensitivity to structural damage, which is associated with local variations of structural material or geometric properties. Such a drawback is particularly significant when dealing with the large scale and complexity of sluice structural systems. To this end, a damage detection method in sluice hoist beams is proposed that relies on the utilization of the local primary frequency (LPF), which is obtained based on the swept frequency excitation (SFE) technique and local resonance response band (LRRB) selection. Using this method, the local mode of the target sluice hoist beam can be effectively excited, while the vibrations of other components in the system are suppressed. As a result, the damage will cause a significant shift in the LPF of the sluice hoist beam at the local mode. A damage index was constructed to quantitatively reflect the damage degree of the sluice hoist beam. The accuracy and reliability of the proposed method were verified on a three-dimensional finite element model of a sluice system, with the noise resistance increased from 0.05 to 0.2 based on the hammer impact method. The proposed method exhibits promising potential for damage detection in complex structural systems.

## 1. Introduction

A sluice is considered as a typical structural system that is frequently used in the field of water infrastructure engineering. To meet the demand of water conservancy functions such as flood control, irrigation, drainage, water diversion, and environmental protection, a great number of sluices have been constructed [1,2]. Hoist beams are important structural components in sluice systems, bearing significant dynamic loads when the sluice gates are opened. Unfavorable factors such as rain erosion, material aging, and over-load will inevitably result in damage occurrence in the hoist beams [3]. The accumulation of damage areas during operation seriously impairs the structural performance and may eventually lead to catastrophic failure [4,5]. Therefore, it is an urgent requirement to develop effective damage detection methods for hoist beams to guarantee the operational safety of sluice structural systems.

Traditionally, damage detection in hoist beams relies heavily on visual inspection, which is largely influenced by subjective factors [6,7,8]. In the past decade, several emerging methods have been proposed to overcome the defects of the visual method [9]. While the methods relying mainly on ground-penetrating radar, ultrasonic, or infrared imaging show advantages of objectivity over the traditional visual inspection method, they have noticeable limitations. For example, ground-penetrating radar is only suitable for detecting damage on large-scale structures. Ultrasonic detection cannot perceive closed cracks that have an insignificant effect on ultrasonic propagation. The infrared imaging method is effective only when the damage position is known. In addition, these methods depend on localized or manual measurement, the efficiency of which is restricted in real-time global information collection [10,11].

Vibration-based methods offer attractive solutions for damage detection in hoist beams [12,13]. The principle of vibration-based methods relies on the fact that damage will alter structural modal parameters [14,15], which, in turn, can reflect the damage state [16,17,18]. Generally, the changes in modal parameters are obtained by conducting field experiments, where the vibration responses of the sluice are measured combined with experimental modal analysis procedures [19,20,21,22]. In theory, rich structural information related to damage positions is contained in mode shapes [23]. However, the measured modal shapes are normally incomplete, even when using multiple sensing positions [24]. On the other hand, the damping ratio is difficult to measure and susceptible to factors such as the ambient temperature, which undermines the reliability of damage detection results [25]. Comparatively, natural frequencies can be measured with high accuracy and stability and thus show promising potential in engineering applications [26,27].

Natural frequency-based damage detection has been widely reported, with significant progress achieved in recent decades [28,29,30]. Chondros et al. studied the relationship between natural frequency changes and crack depths in a fixed beam based on theoretical analysis and experiments [31,32]. Chinchalkar et al. simulated cracks using rotating springs and studied the effect of the damage size and position on natural frequencies. The first three-order natural frequencies were used to identify damage positions [33]. Wei et al. proposed a damage detection method based on the natural frequency vector and its guarantee criterion. The numerical results demonstrated that the method can accurately identify the damage position and degree in a simply supported beam [34]. Wang et al. investigated the feasibility and effectiveness of using the acceleration frequency function to identify the structural damage in underground tunnel structures [35]. Wang et al. proposed a frequency-based method to describe the dynamic response of a structure with an auxiliary mass combined with information of frequency shifting and amplitude changing [36]. Nevertheless, the sensitivity of existing methods based on natural frequency is not sufficient for local damage in complex, large-scale structures such as sluice structural systems [37,38,39]. Therefore, it is crucial to effectively reflect the local damage in the global complex structure.

This study attempted to develop a damage detection method for hoist beams based on structural local modes. In structural dynamics, a local mode is defined as a specific mode for a complex structural system consisting of multiple substructures, of which one substructure vibrates, overwhelming other substructures. Such a substructure absorbs almost all of the vibration energy, and the damage effect on this substructure will be significantly magnified [40,41]. Mei et al. utilized local vibration under the excitation of piezoelectric wafer active sensors to detect and quantify delamination in composite plates [42]. Hou et al. defined the natural frequency corresponding to the local mode as the local primary frequency (LPF) of the substructure. Due to the high sensitivity of the LPF to damage, a single-order LPF is sufficient for damage identification in the substructure [43]. The premise of using LPF to detect damage in the hoist beams is to fully stimulate the local mode, which depends on suitable excitation methods. This study proposed a method of using swept frequency excitation (SFE) combined with local resonance response band (LRRB) selection to obtain the local modes of a sluice system. This work is a different attempt from other methods, especially those focusing on algorithms, and is expected to break through the shortcomings of existing methods from another perspective. The innovation of this method lies in the ingenious excitation and use of the local mode phenomenon to overcome the insensitivity of frequency to damage, thus facilitating damage detection.

The rest of this paper is organized as follows: Section 2 theoretically demonstrates that the LPF mainly reflects the vibration characteristics of the substructure by the frequency sensitivity to substructure damage. Section 3 provides a numerical example of a fixed beam to illustrate the realization of the proposed method with the assistance of the LRRB. Section 4 constructs the numerical model of a sluice system to verify the effectiveness of the method. Section 5 carries out a parametric study to discuss the influencing factors such as the sensor position, excitation form, and measurement noise, and Section 6 presents the conclusions.

## 2. Damage Factor Formation Based on Local Modes

### 2.1. Frequency Sensitivity to Substructural Damage

For an n DOF linear system comprising m substructures, the vibration characteristic equation of the system can be expressed as

(1) $$\left(K\;-\;\omega_{r}^{2}M\right)\varphi_{r}=0$$

where **K** and **M** are the global stiffness and mass matrix, respectively; $\omega_{r}$ and $\varphi_{r}$ represent the rth-order angular frequency and modal shape, respectively. $\alpha_{i}$ is defined as the coefficient of stiffness reduction caused by damage on the ith substructure, and $K_{i}^{e}$ is the stiffness matrix of the substructure. Hence, the global stiffness matrix can be integrated to be

(2) $$K=\sum_{i=1}^{m}\alpha_{i}K_{i}^{e}$$

Generally, the mass matrix is assumed to be irrelevant with damage, and the local stiffness matrix of the jth (j ≠ i) substructure is also not affected by damage existence; thus, it has

(3) $$\frac{\partial M}{\partial \alpha_{i}}=0;\frac{\partial K_{j}}{\partial \alpha_{i}}=0$$

The frequency sensitivity coefficient can be defined as

(4) $$\xi_{r,i}=\frac{\partial \omega_{r}}{\partial \alpha_{i}}=\frac{\varphi_{i,r}^{T}K_{i}^{e}\varphi_{i,r}}{2\omega_{r}}$$

For a given system, the frequency sensitivities of different substructures are compared at the same order of frequency $\omega_{r}$, and $K_{i}^{e}$ can be constructed, meaning $\xi_{r,i}$ is dependent on the magnitudes of the modal shapes.

### 2.2. Local Modes of Substructures

For an n DOF system (comprising m substructures), a series of $\xi_{r,i}$ (1< r < n) can be constructed according to Equation (4) for damage identification on substructure i. Assuming the highest $\xi_{r,i}$ magnitude, corresponding to the vth vibration mode, is

(5) $$\xi_{v,i}=\frac{\varphi_{i,v}^{T}K_{i}^{e}\varphi_{i,v}}{2\omega_{v}}$$

$\xi_{v,i}$ is usually associated with a local mode of the system, where the response amplitude of the ith substructure is the highest among all substructures, and the corresponding natural frequency is defined as the local primary frequency, i.e., $\omega_{\mathrm{LP}}$ [40]. Under the local mode, a large amount of structural vibration energy is concentrated on the ith substructure, the damage in which will, in turn, cause significant variation in $\omega_{\mathrm{LP}}$.

### 2.3. Damage Index

Leveraging on the high damage sensitivity of local modes, the change ratio of $\omega_{\mathrm{LP}}$ can be utilized to indicate the existence and severity of damage, giving rise to a damage index defined as

(6) $$\mathrm{DI}=\frac{\omega_{\mathrm{LP},n}-\omega_{\mathrm{LP},d}}{\omega_{\mathrm{LP},n}}$$

where $\omega_{\mathrm{LP},n}$ and $\omega_{\mathrm{LP},d}$ are captured under undamaged and damaged states of structures, respectively. In theory, the local mode and $\omega_{\mathrm{LP}}$ can be identified by solving the characteristic equation of vibration and by mode shape comparison. However, it is difficult to obtain the local modes in engineering practice because of the uncertainties associated with factors such as material/geometric parameters and boundary conditions.

## 3. Damage Identification Evoked by Swept Frequency Excitation

### 3.1. Swept Frequency Excitation (SFE)

**Figure 1:**
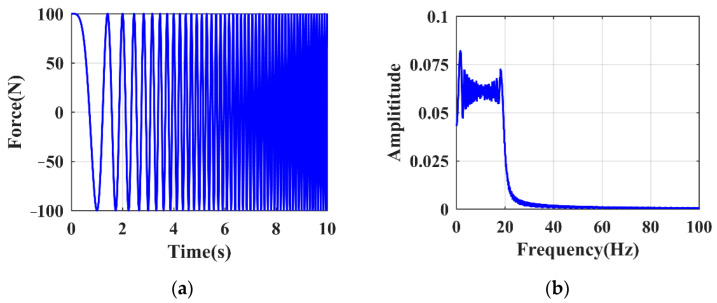
Typical swept frequency excitation: (**a**) time domain; (**b**) frequency domain.

For linear systems, vibration characteristics are often characterized using the vibration amplification coefficient by referring to static displacement. Defining the frequency ratio as β = ω/$\omega_{\mathrm{LP}}$, the amplification coefficient is expressed as

(7) $$\lambda =\frac{1}{\sqrt{{{(1\;-\;\beta }^{2})}^{2}{+(2\xi \beta )}^{2}}}$$

where ξ is the damping ratio; ω is the frequency of excitation. For a structure with a given damping ratio, ω = $\omega_{\mathrm{LP}}$ is able to generate the local mode with maximized λ. In contrast, λ is minimal when ω and $\omega_{\mathrm{LP}}$ are largely different in frequency. To accurately identify $\omega_{\mathrm{LP}}$ by taking into account structural uncertainties, SFE is an appropriate means to generate the expected responses and prevent misleading results. Compared to hammer excitation (HE), SFE is much more controllable and able to generate excitation energies distributed widely and uniformly in the frequency domain, from which the expected local modes can be accurately extracted. Specifically, SFE is performed using sinusoidal excitation signals with a continuously varied frequency [44]. The range and transformation mode of the frequency are the two main parameters considered in SFE [45], where the linear transformation mode is commonly applied. Figure 1 shows a typical SFE waveform with the linear transformation mode ranging between 0 and 25 Hz.

### 3.2. Local Resonance Response Band (LRRB)

**Figure 2:**
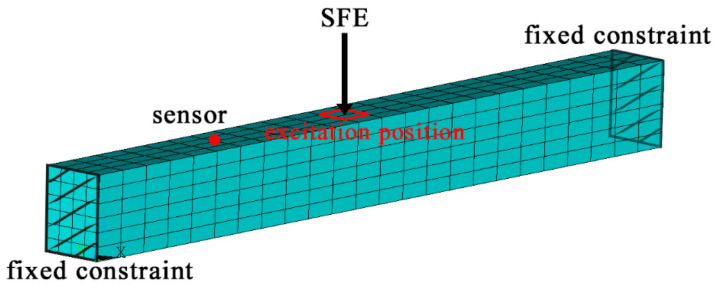
Finite element model of a two-end fixed beam.

Local mode identification based on SFE was applied to a two-end fixed beam for illustration. The beam can be regarded as a substructure in a structural system containing multiple components. The sectional dimensions of this fixed beam are 0.60 m in width, 0.80 m in height, and 8.0 m in span. The elastic modulus and damping ratio of this fixed beam are 3.1E10 Pa and 0.05, respectively. A finite element (FE) model of the beam was built using solid elements in the commercial software ANSYS/APDL, as shown in Figure 2. SFE was applied at the excitation position with a frequency band ranging from 0 to 250 Hz, and acceleration responses were collected subjected to the SFE at the sensor location.

**Figure 3:**
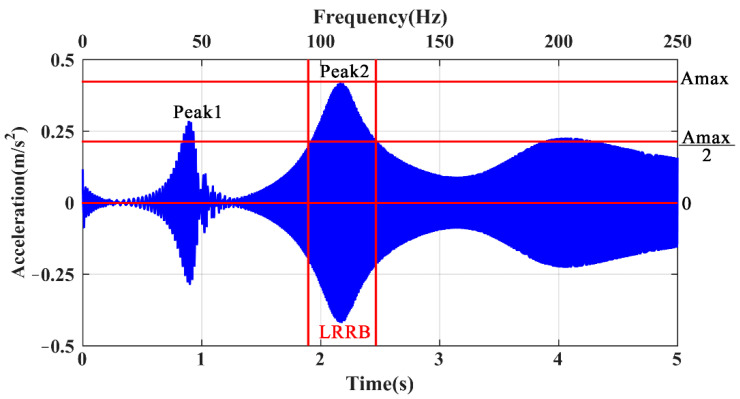
Typical response of a two-end fixed beam in the time domain.

A total of 10,000 sampling points were collected within 5 s at a sampling frequency of 2000 Hz. Two resonance peaks in the response signals can be seen within the time period, as shown in Figure 3. The excitation frequencies at these two peaks are close to the theoretical modal frequencies of 49.13 and 109.43 Hz, which can be calculated according to modal analysis. In applications, the local modes of a structural system can be identified according to such resonance, measured on a substructure, the responses of which are much more significant than those of other substructures. The responses close to the resonance peaks are then used for damage assessment. In this study, the response areas between the maximum and half amplitudes are defined as the LRRB, as illustrated in Figure 3.

### 3.3. Procedure of Damage Identification

**Figure 4:**
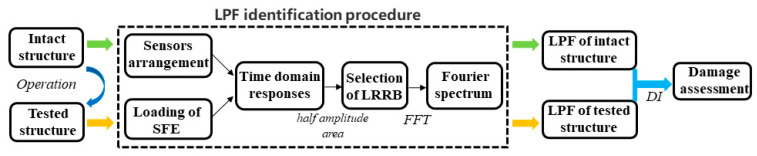
Flowchart of the proposed method.

The procedure of damage identification is shown in Figure 4: (1) arrange the positions of sensors and load SFE excitations; (2) identify the LRRB from the time domain responses based on the half amplitude area; (3) transform the LRRB section by FFT and identify the LPF in the frequency domain; (4) quantify the damage based on the shift in the LPF according to Equation (6).

## 4. Application to Evaluation of Sluice Beam

### 4.1. Finite Element Model

**Figure 5:**
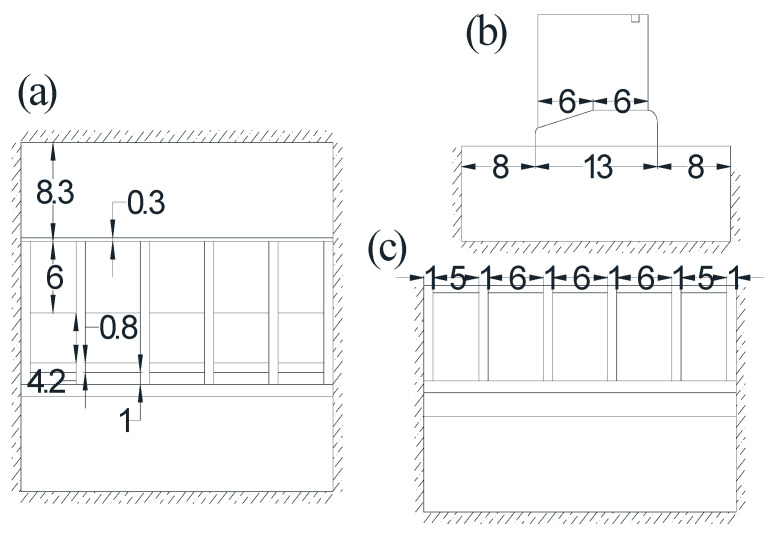
Geometry of the sluice: (**a**) plane view; (**b**) cross-section view; (**c**) elevation view.

**Figure 6:**
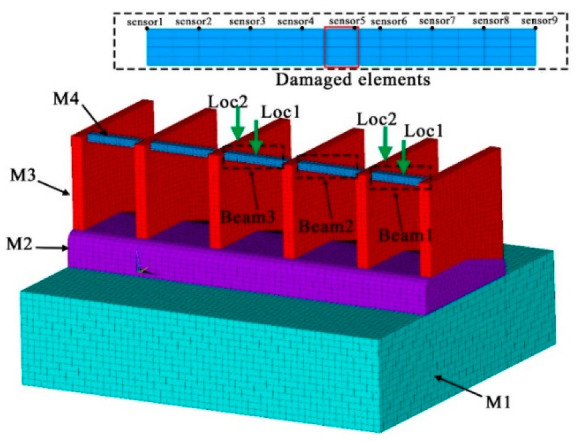
Finite element model of the sluice system.

In this section, a numerical model of a five-hole sluice is built to verify the proposed method. The model is composed of the foundation, the baseplate, the gate pier, and the hoist beams. The dimensions of the sluice are illustrated in Figure 5 (unit: m), and the FE model of the sluice is constructed using 8-node solid elements and 4-node tetrahedral elements, as presented in Figure 6. The bottom surface of the foundation is fully constrained, and the upper and lower surfaces and the left and right surfaces of the foundation are normally constrained. Table 1 shows the material properties of the sluice.

### 4.2. Simulation of Damage

Although multiple types of damage may coexist in the sluice, single damage is most likely to occur at the early service stage and is thus crucial to be detected accurately. Specifically, mid-span damage in the hoist beams is elaborated by reducing the elastic modulus of the damaged elements to mimic various degrees of damage, as shown in Figure 6. Due to structural symmetry, only three beams are assumed to contain damage. All damage cases are numbered in Table 2, where the ones with a relative amount of stiffness reduction from 5 to 50%, with a uniform increment of 5%, are used for the construction of the reference data, and the damage cases with a stiffness reduction of 12, 27, and 47% are to be identified quantitatively.

### 4.3. Damage Identification

**Figure 7:**
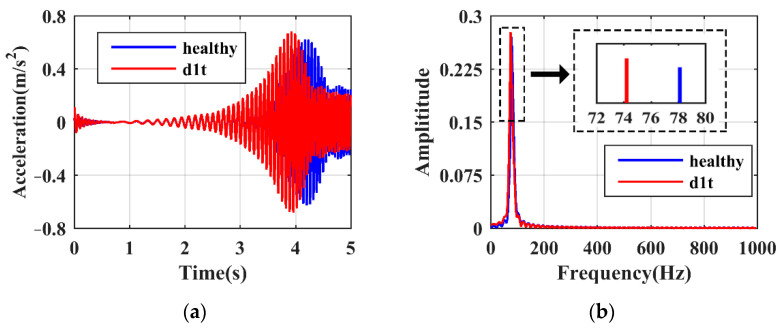
Damage characteristics of case ‘d1t’: (**a**) time domain; (**b**) frequency domain.

**Figure 8:**
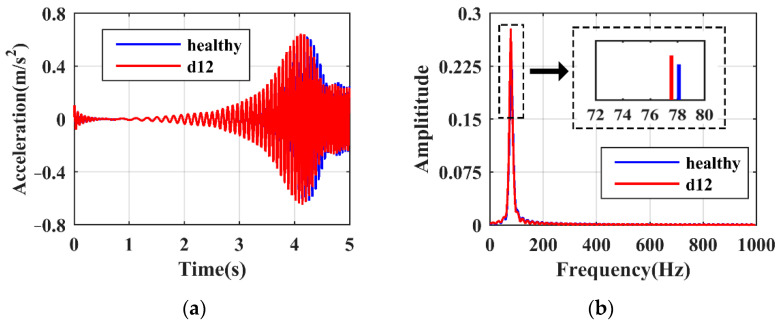
Damage characteristics of case ‘d12′: (**a**) time domain; (**b**) frequency domain.

Subjected to SFE, the responses of the hoist beams under healthy and damaged states are firstly shown. Figure 7a,b present the time and frequency domain responses of case ‘d1t’, respectively. It is seen in Figure 7a that the resonance peak shifts to the left due to damage. The time domain signals within the LRRB are then selected and transformed using the FFT algorithm, giving rise to the frequency domain responses shown in Figure 7b, where the values of $\omega_{\mathrm{LP}}$ can be identified. Due to damage existence, $\omega_{\mathrm{LP}}$ apparently decreases from 78.13 to 74.22 Hz. On the other hand, the vibration responses under ‘d12′, which represents a relatively slight damage case, are shown in Figure 8. Although the response variations in the time and frequency domains are not as obvious as in ‘d1t’, the change of $\omega_{\mathrm{LP}}$ from 78.13 to 77.58 Hz can still be recognized. It should be noted that the responses obtained at local modes have a strong immunity to the influence from other substructures. Therefore, damage detection using SFE and LRRB is able to achieve high reliability.

**Figure 9:**
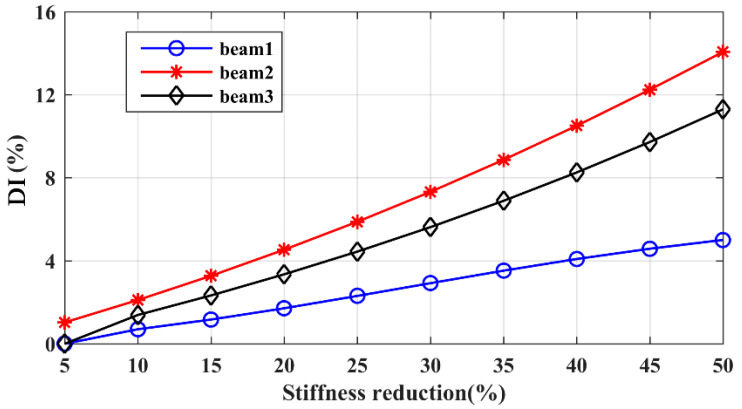
Damage curves: beam 1; beam 2; beam 3.

Figure 9 shows the results of damage identification in different cases, as presented in Table 2. In general, the DI values (in Equation (6)) increase linearly along with an enlarged damage severity, and it should be noticed that the three substructures are independent in the variation in DI without mutual interference, benefiting from the adoption of local modes. Based on the reference data in Figure 9, DI values are calculated to predict the existence and degree of the nine damage cases in Table 2. The identification results are presented in Table 3. The averaged and maximum errors between the identified and true values are 1.59 and 4.17%, respectively, indicating a satisfactory accuracy of the proposed method in estimating the damage severity in hoist beams. This method still works well even in small damage cases of around 10%, which means that the method has the ability to detect original damage.

## 5. Parametric Discussions and Comparison with Hammer Excitation (HE) Method

### 5.1. Sensor Position

**Figure 10:**
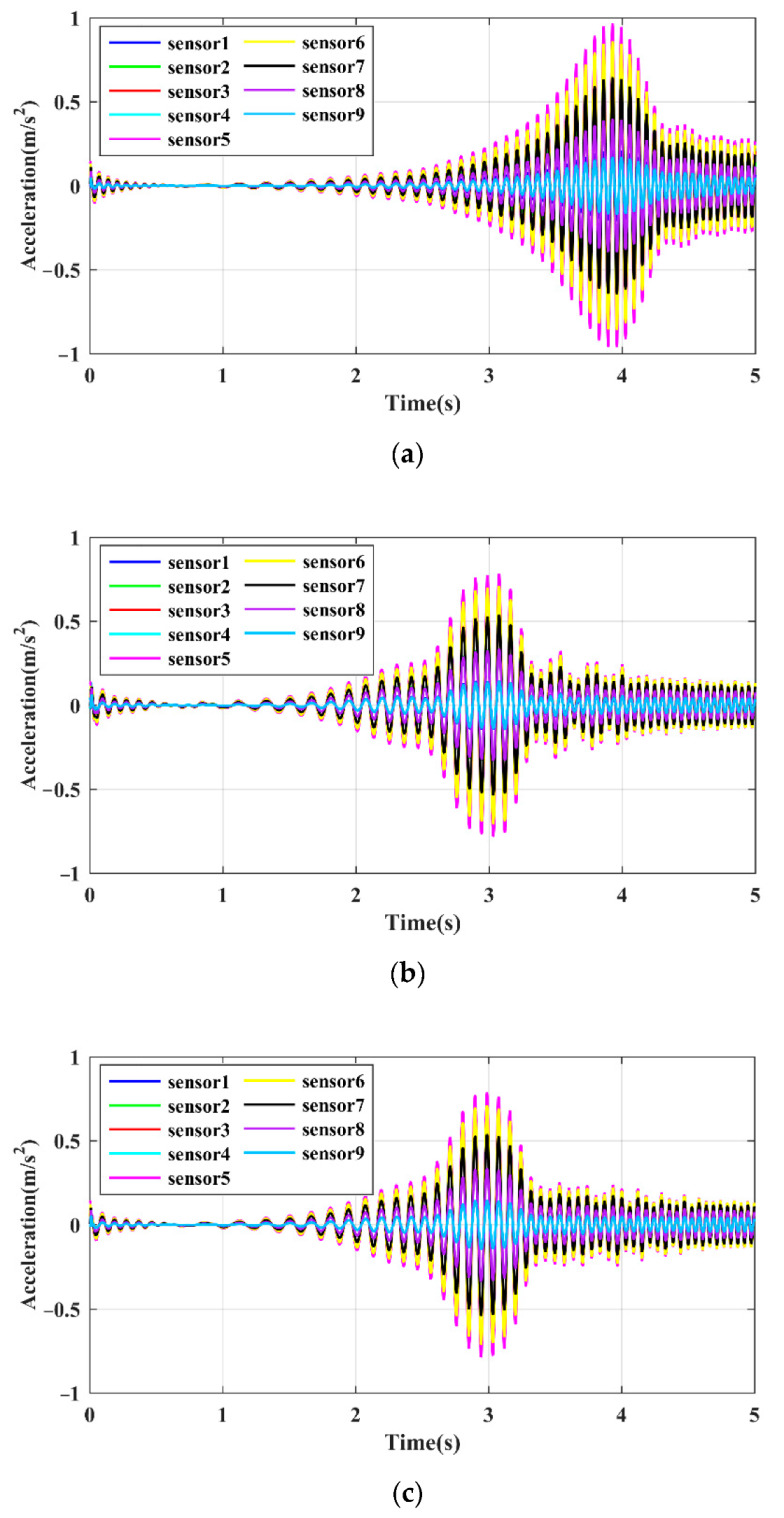
Time domain responses measured by different sensors: (**a**) beam 1; (**b**) beam 2; (**c**) beam 3.

**Figure 11:**
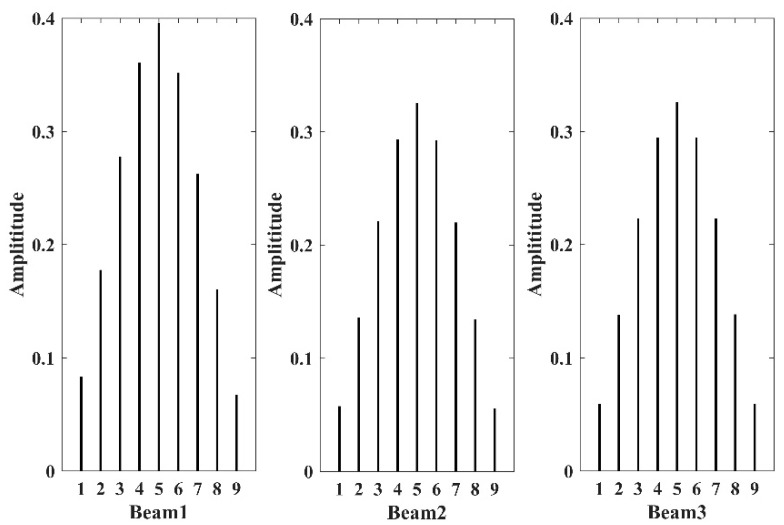
The amplitudes of frequency domain responses of the LRRB measured by different sensors under different damage situations.

The effect of the sensor position was examined according to the responses of different FE nodes, as shown in Figure 6, where the three beams to be studied have the same sensor arrangement. Using the damage cases of d1t, d2t, and d3t as examples, the time domain responses at the nine sensor positions are shown in Figure 10. The resonance peaks associated with different sensor positions do not show a difference in time but in amplitude. Only five responses are distinguishable due to the symmetry in the geometry of the hoist beams. The response amplitudes measured by the sensors near the middle span of the beam are the highest and are deemed proper to be used for damage identification. The frequency domain identification results by using the LRRB under different damage conditions are shown in Figure 11. Similar to the time domain response, there are several peaks with significantly different amplitudes at different measurement positions. Therefore, the position of the sensor has no effect on the obtained frequency value but has a significant effect on the amplitude of the time domain responses and LRRB. The sensor should be arranged in the position with a larger vibration amplitude as far as possible to improve the signal quality, and the middle of the span should be selected for beam-type structures to be detected.

### 5.2. Excitation

**Figure 12:**
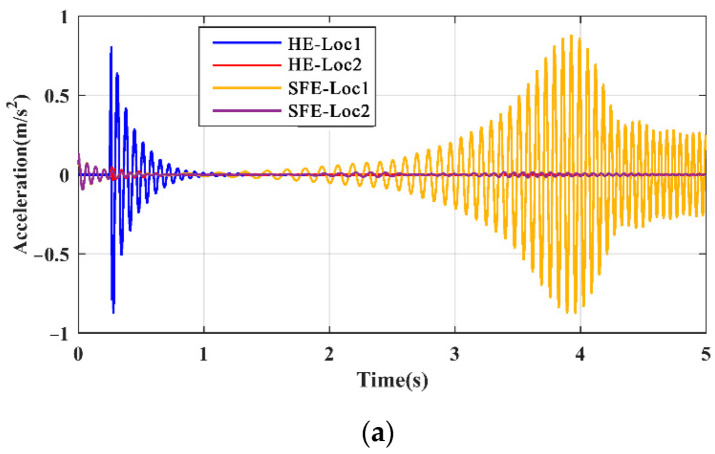

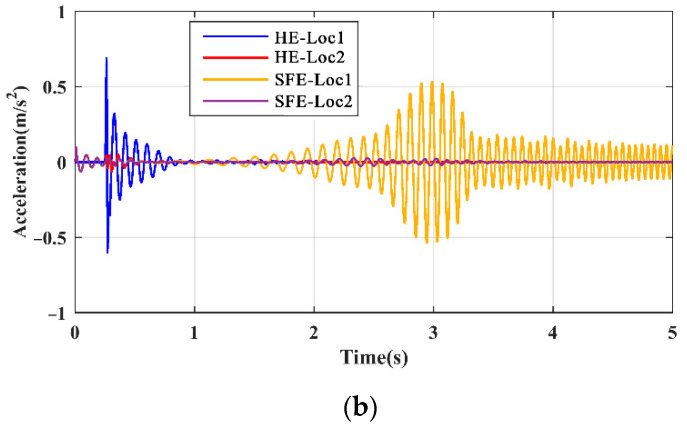
Time domain responses excited by different excitations: (**a**) beam 1; (**b**) beam 3.

The effect of the form and position of the excitation on the local mode was studied. Since there is no significant difference between the responses of beam 2 and beam 3, only beam 1 and beam 3 were investigated. The positions of the excitation are denoted as Loc1 on the beam and Loc2 on the gate pier, and the forms of the excitation are denoted as SFE and HE, respectively. The responses of beam 1 and beam 3 under four excitation situations, i.e., HE-Loc1, HE-Loc2, SFE-Loc1, and SFE-Loc2, are presented in Figure 12. Under the excitation of HE, the response decays rapidly due to energy absorption caused by structural damping. Under the excitation of SFE, the response of the structure is related to the frequency of the excitation varying in the time domain. As for the excitation position, the response amplitudes obtained by direct excitation on the beam are larger, under both the SFE and HE situations, compared with those on the gate pier.

**Figure 13:**
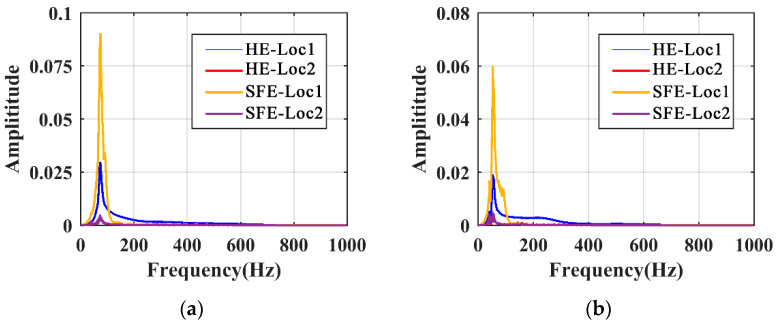
Frequency domain responses excited by different excitations: (**a**) beam 1; (**b**) beam 3.

From the frequency domain perspective, the spectrum is obtained by the FFT of the original signal transformation, rather than the LRRB signal, because there is no presence of the LRRB for HE, as shown in Figure 13. Although SFE and HE obtain an equal LPF, the response amplitudes measured by SFE are larger because it provides a more sufficient input energy to the structures. The responses measured on Loc2 are minimal in magnitude, which means it is necessary to directly excite the substructure of interest.

### 5.3. Noise Immunity

The signals measured in actual practice are inevitably interfered with by noise originating from the measurement system or environment. Identifying LPFs under noisy conditions is crucial in the implementation of the proposed method. To study the noise robustness, different levels of Gaussian noise are added to the time domain acceleration signals according to

(8) $$S_{\mathrm{noisy}}{\;=\;S}_{\mathrm{clean}}\;+\;\sigma \;*\;\mathrm{randn}\left(N,1\right)$$

where $S_{\mathrm{noisy}}$ is the signal polluted by noise, $S_{\mathrm{clean}}$ is the original signal without a noise influence, σ is the intensity of the noise signal, and N is the length of the signals.

**Figure 14:**
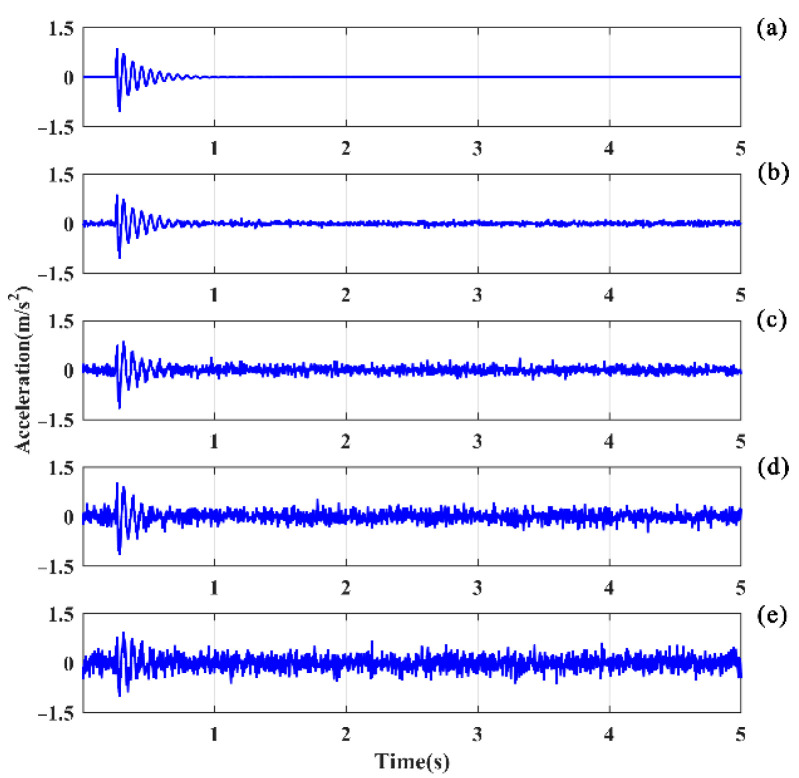
Clean signals and noisy signals in the time domain of beam 1 excited by HE: (**a**) σ = 0; (**b**) σ = 0.05; (**c**) σ = 0.10; (**d**) σ = 0.15; (**e**) σ = 0.20.

**Figure 15:**
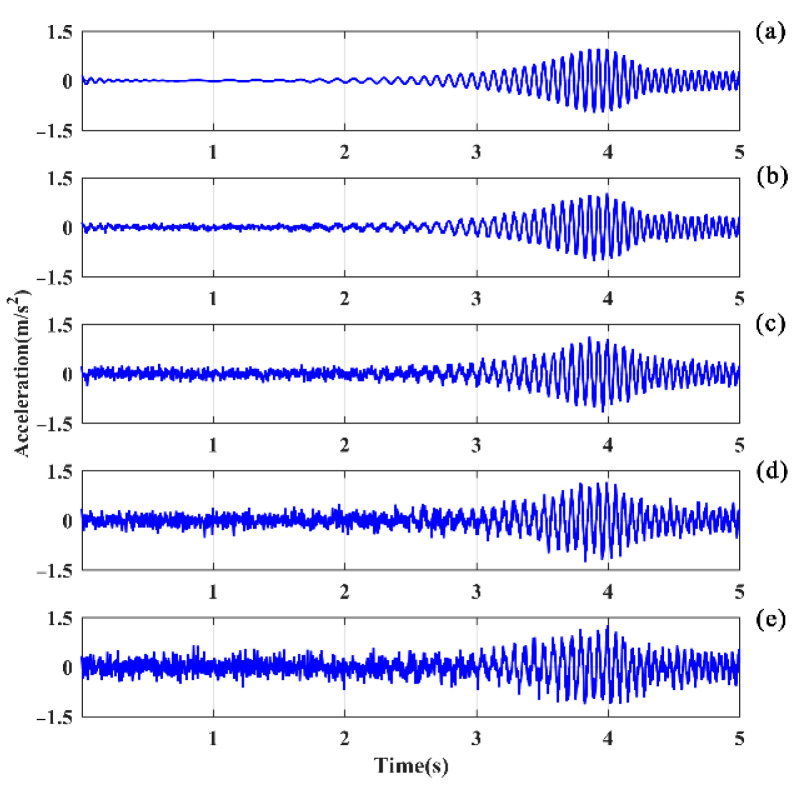
Clean signals and noisy signals in the time domain of beam 1 excited by SFE: (**a**) σ = 0; (**b**) σ = 0.05; (**c**) σ = 0.10; (**d**) σ = 0.15; (**e**) σ = 0.20.

**Figure 16:**
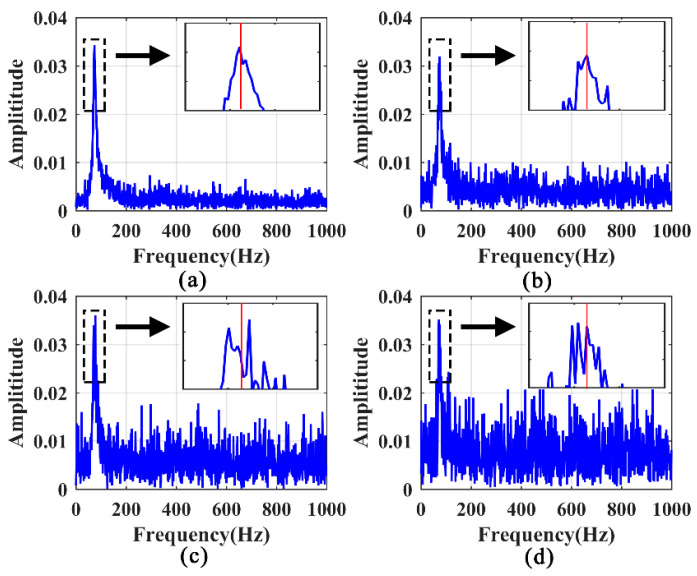
Identification of LPF in frequency spectrums of noisy signals excited by HE: (**a**) σ = 0.05; (**b**) σ = 0.10; (**c**) σ = 0.15; (**d**) σ = 0.20.

The responses associated with excitations applied on the hoist beams were considered, the responses at the middle span of beam 1 with a 50% damage degree were extracted, and the HE and SFE signals are shown in Figure 14 and Figure 15, respectively. For HE, the LPF peak remains prominent at a small noise level, as shown in Figure 16a. With σ = 0.1, the LPF can still be identified through the spectrogram, although a disturbing peak appears adjacent to the true LPF peak. With the increase in noise, the LPF cannot be identified exactly because of the disturbing peaks occurring beside the real LPF peak, as it can be seen in Figure 16c,d.

**Figure 17:**
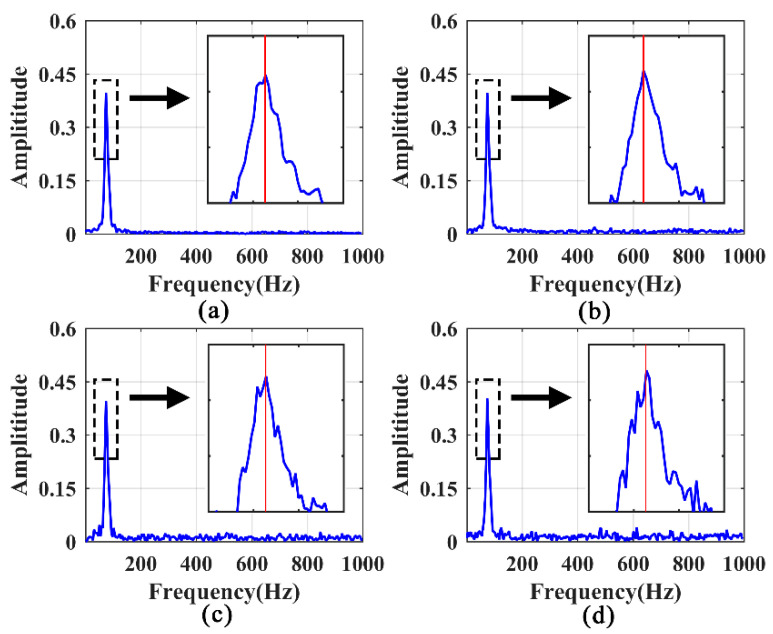
Identification of LPF in frequency spectrums of noisy signals excited by SFE: (**a**) σ = 0.05; (**b**) σ = 0.10; (**c**) σ = 0.15; (**d**) σ = 0.20.

Comparatively, the noise robustness of SFE is superior to that of HE. As shown in Figure 17, the interference of noise is insignificant compared to the noise-free signal, and the LPF can be accurately identified.

## 6. Conclusions

To address the issue of the insufficient sensitivity of frequency changes to local structural damage, a method for detecting damage in sluice hoist beams was proposed based on the LPF of substructures. Numerical simulation of a five-hole sluice demonstrated that in the complex sluice structural system, local modes of substructures can be excited and utilized by adjusting the excitation forms and positions. The corresponding LPF was mainly related to the dynamic characteristics of the substructure, and the sensitivity of the LPF to damage in the substructure was high. Specifically, the damage degree of the sluice hoist beam was correlated with the change ratio of the LPF. Relying on reference data constructed under several damage cases, the arbitrary damage degree can be identified using DI with errors limited within an acceptable range. In the ideal condition, the identified frequency value is independent of the sensor positions. However, the sensor positions at the middle span of the hoist beams are able to obtain high-amplitude responses suitable for damage identification.

Compared with the most widely used hammer impact method, this method inputs more energy into the sluice structure and has better controllability, which makes it easier for the operator to obtain the LPF. In particular, the proposed method exhibits greater noise immunity considering that the actual operation is interfered with by noise. According to the defined noise intensity, this method increased the noise resistance from 0.05 to 0.2 based on the hammer impact method. The proposed method applies a frequency index which reflects the global character locally in the substructure and exhibits promising potential in damage detection applications for large-scale complex structural systems. This work preliminarily evaluated the damage degree based on a simple and practical damage index, and the combination of local modes with other damage indicators will be the continuation of this work.

## Figures and Tables

**Table 1:** Material parameters of the sluice system.

Name	M1	M2	M3	M4
Type	Foundation	Bottom plate	Gate pier	Hoist beam
Density (Kg/m^3^)	2450	2650	3100	2650
Elastic Modulus (MPa)	5E3	2.8E4	3.1E4	3E4
Poisson’s Ratio	0.28	0.167	0.167	0.2

**Table 2:** Damage situations for sluice hoist beams.

Reduction (%)	5	10	15	20	25	30	35	40	45	50	12	27	47
Beam 1	d11	d12	d13	d14	d15	d16	d17	d18	d19	d1t	T1a	T1b	T1c
Beam 2	d21	d22	d23	d24	d25	d26	d27	d28	d29	d2t	T2a	T2b	T2c
Beam 3	d31	d32	d33	d34	d35	d36	d37	d38	d39	d3t	T3a	T3b	T3c

**Table 3:** Damage identifications results.

Damaged Cases	T1a	T1b	T1c	T2a	T2b	T2c	T3a	T3b	T3c	Average
True results (%)	12	27	47	12	27	47	12	27	47	/
Identified results (%)	12.1	26.5	46.3	11.6	27.1	47	12.5	27.4	46.6	/
Error (%)	0.83	1.85	1.49	3.33	0.37	0	4.17	1.48	0.85	1.59

## Data Availability

Not applicable.
